# Cross-cultural adaptation and validation of the Northwick park neck pain questionnaire to Urdu language

**DOI:** 10.1186/s12891-023-06586-5

**Published:** 2023-06-05

**Authors:** Muhammad Nazim Farooq, Somiya Naz, Ambrin Kousar, Komal Shahzad

**Affiliations:** Islamabad College of Physiotherapy, Margalla Institute of Health Sciences, Quaid-e-Azam Avenue Gulrez III, Rawalpindi, Pakistan

**Keywords:** Neck pain, Northwick Park Neck Pain Questionnaire, Outcome assessment, Reliability, Responsiveness, Translations, Validity

## Abstract

**Background:**

Despite its widespread use for assessing pain and disability in patients suffering from neck pain, the Northwick Park Neck Pain Questionnaire (NPQ) has yet to be translated and validated in Urdu. The purpose of the present study was to translate and cross-culturally adapt the NPQ into Urdu language (NPQ-U), and to investigate the NPQ-U’s psychometric properties in patients with non-specific neck pain (NSNP).

**Methods:**

The NPQ was translated and cross-culturally adapted into Urdu in accordance with the previously described guidelines. The study included 150 NSNP patients and 50 healthy participants. The NPQ-U, Urdu version of neck disability index (NDI-U), neck pain and disability scale (NPDS), and numerical pain rating scale (NPRS) were completed by all participants on first visit. After three weeks of physical therapy, the patients completed all of the questionnaires listed above, along with the global rating of change scale. Test-retest reliability was determined on 46 randomly selected patients who completed the NPQ-U again two days after the first response. The NPQ-U was evaluated for internal consistency, content validity, construct (convergent and discriminative) validity, factor analysis, and responsiveness.

**Results:**

The NPQ-U demonstrated excellent test-retest reliability (intra-class correlation coefficient = 0.96) and high internal consistency (Cronbach’s alpha = 0.89). There were no floor or ceiling effects for the NPQ-U total score, indicating good content validity. A single factor was extracted, which explained 54.56% of the total variance. For convergent validity, the NPQ-U showed a strong correlation with NDI-U (r = 0.89, P < 0.001), NPDS (r = 0.71, P < 0.001), and NPRS (r = 0.73, P < 0.001). The results revealed a significant difference between patients and healthy controls in the NPQ-U total scores (P < 0.001) demonstrating significant discriminative validity. A significant difference in the NPQ-U change scores between the stable and the improved groups (P < 0.001) confirmed its responsiveness. Furthermore, the NPQ-U change score showed a moderate correlation with NPDS change score (r = 0.60, P < 0.001) and NPRS change score (r = 0.68, P < 0.001), but a strong correlation with NDI-U change score (r = 0.75, P < 0.001).

**Conclusion:**

The NPQ-U is a reliable, valid, and responsive tool for assessing neck pain and disability in Urdu-speaking patients with NSNP.

## Introduction

Neck pain has emerged as one of the major global health problems because of its high prevalence, incidence and associated disability [1, 2]. Around 290 million cases of neck pain were recorded worldwide in 2017, with an age-standardized point prevalence of about 36 cases per 1000 people [2]. The annual incidence of neck pain was found roughly 8 cases per 1000 population [2]. Neck pain has been recorded more in females than males [1, 2]. Neck pain is common in various occupational groups in Pakistan, ranging from 26.5 to 72% [3–6]. The observed neck pain burden has been increased remarkably over the past three decades [2]. Neck pain has a substantial economic impact, including treatment costs, lost productivity, and job-related issues. With an estimated $134.5 billion in health-care spending in 2016, low back and neck pain accounted for a large proportion of health-care spending in the United States [7].

In clinical practice, patient-reported outcome measures are commonly used. These outcome measures, according to a recent systematic review, stimulate active patient participation, improve quality of care, boost consultation focus (by prioritizing patient needs), allows for standardized assessment of patient outcomes, and strengthen the patient–clinician relationship [8]. Many researchers emphasized the importance of adapting previously recognized and widely used assessment tools rather than developing a new questionnaire [9–11].

The Neck Disability Index (NDI) [12] and the Northwick Park Neck Pain Questionnaire (NPQ) [13] are commonly used scales for assessing neck pain-related disability. Both questionnaires were adapted from the Oswestry Disability Questionnaire [14] and were designed to be filled out directly by the patient, while also including some common items. The NDI has already been translated, cross-culturally adapted, and validated to Urdu. Despite the fact that the NDI is the most commonly used to assess neck pain-related disability, the NPQ appears to have some advantages because it asks questions about functional limitations that are directly related to neck pain, whereas the NDI asks more generic questions (for example, about ability to lift heavy objects and concentration). The NPQ is simple to administer and score, and it provides an objective measure of outcome in patients with neck pain [13]. This questionnaire has been translated and validated into Turkish [15], Spanish [16], Chinese [17], French [18], Greek [19], Brazilian Portuguese [20], Korean [21], and Hausa [22], and it has shown good psychometric properties in all of these languages. To the best of the authors’ knowledge, the NPQ has not yet been translated and validated in Urdu.

The purpose of this study was to translate and culturally adapt the NPQ into Urdu (NPQ-U) using recognized methodologies, as well as to examine the translated version’s psychometric properties in Urdu-speaking patients with non-specific neck pain (NSNP).

## Materials and methods

### Translation and cross-cultural adaptation

After receiving consent from the developer of the original NPQ, the translation and cultural adaption processes were initiated. These procedures were carried out in accordance with the COSMIN (COnsensus-based Standards for the selection of health status Measurement INstruments) criteria and the previously mentioned standards [10, 23]. There were five steps in the entire procedure.

#### Step I

The NPQ was independently translated from English into Urdu by two native Urdu-speaking translators who were also fluent in English. One of the translators was an English professor, and the other was a senior lecturer in physiotherapy. Both translators were given the instruction to translate conceptually rather than literally. Both of them provided written reports.

#### Step II

By combining the findings of the two translated versions and resolving differences, the translators and two researchers created a consensus version.

#### Step III

Two professional translators blinded to the original version translated the agreed-upon Urdu version back into English. Both translators were unfamiliar with the questionnaire concept, and neither had any medical background.

#### Step IV

An expert committee comprised of researchers, translators, an assistant professor of Physiotherapy, and a methodologist reviewed all translations, the consensus version, and the original questionnaire. A pre-final NPQ-U was obtained after reaching agreement on idiomatic, semantic, experiential, and conceptual equivalence.

#### Step V

Forty patients with NSNP were recruited to test the face validity of the NPQ-U’s pre-final version [10]. The patients were asked to fill out the questionnaire. Following that, each item on the questionnaire was discussed with the patients one by one. Patients were asked to express their understanding of each question and its responses, as well as their perceptions of the items’ relevance to their situation and their ability to complete the questionnaire independently. Additionally, patients were encouraged to report any issues with the questionnaire’s instructions, wording, or layout. The expert committee assessed all findings from this stage of the adaptation process, and after reaching a consensus, the final NPQ-U was created.

### Instruments

#### NPQ

The NPQ assesses neck pain and the resulting patient disability [13]. It consists of nine questions. Each question has five responses. The questions are related to the intensity of neck pain, pins and needles/numbness, the duration of the symptoms, and various physical activities (carrying, sleeping, reading/watching television, social activities, work, and driving). Each item’s score ranges from 0 to 4. The maximum total score possible is 36, which is converted to a percentage. Higher scores indicate greater disability. A tenth question is added at the follow-up to assess the patient’s current pain status in comparison to the last time the NPQ was completed. It’s simple to complete, easy to score, and has good psychometric properties [13, 24].

#### Neck pain and disability scale (NPDS)

The NPDS, which consists of 20 items, is one of the most widely used neck pain-related disability scales [25]. Each item is graded from 0 to 5 on a 100-mm visual analogue scale. The total NPDS score ranges from 0 (no disability) to 100 (the most severe disability). The NPDS has proven to be a valid and reliable tool [24, 25].

#### Urdu version of NDI (NDI-U)

The NDI-U consists of ten items, each with six possible responses [26]. Each item’s score ranges from 0 to 5. The highest possible total score is 50, which is converted to a percentage. Higher scores indicate greater disability. The NDI-U has been shown to be a valid, reliable, and responsive questionnaire for patients suffering from neck pain [26].

#### Numerical pain rating scale (NPRS)

The NPRS is an 11-point scale that ranges from 0 (no pain) to 10 (worst pain imaginable) and is used to assess the patient’s pain intensity. Patients were asked to rate their neck pain by selecting a number on the scale that best represented their pain level over the previous 24 h. The NPRS has been demonstrated to be a reliable and valid tool for assessing pain intensity [27].

#### Global rating of change (GROC) scale

The GROC scale, having good reproducibility and sensitivity to change, is widely used to assess subject’s progress and deterioration over time usually to determine the outcome of an intervention. The outcomes were determined by asking the patients to mark a number from − 7 (“a very much worse”) to + 7 (“a very much better”) that represented their current health condition after recalling their condition at the start of the treatment [28].

### Psychometric testing

Psychometric testing of the NPQ-U was performed according to COSMIN guidelines [23].

### Participants

Both male and female patients with age 18–65 years having NSNP and able to read Urdu were recruited from two hospitals and one Rehabilitation Centre of Islamabad and Rawalpindi, Pakistan through convenience sampling technique. To calculate sample size for general psychometric testing, ten subjects per item of instrument are required [29]. Despite the fact that the calculated sample size for this study was 90 due to the nine items on the NPQ-U, 150 patients were recruited for this study to achieve the large sample size recommended by previous guidelines [11]. Patients were excluded if they had neck pain related to vertebral fracture, myelopathy, neck/brain surgery, infectious/inflammatory diseases, neurological deficits, tumors, or other systemic diseases. Patients with diagnosed psychiatric disorders were also excluded. Moreover, fifty healthy participants aged 18–65 years having no history of pain or neck pathology were also recruited from the students and staff of the Margalla Institute of Health Sciences Rawalpindi. The study was conducted from August 2019 to June 2021.

### Procedure

A self-structured questionnaire was used to collect demographic and disease-related information. Both patients and healthy controls were asked to fill the NPQ-U, NPDS, NDI-U, and NPRS on day first. Then out of 150 patients, 46 randomly selected patients were asked to fill the NPQ-U again following 48 h of the first response. Patients received physical therapy treatment for 3 weeks and after 3 weeks, patients were asked to fill the NPQ-U, NPDS, NDI-U, NPRS, and GROC scale. All participants provided written informed consent. This study was approved by the ethics review committee of Margalla Institute of Health Sciences, Rawalpindi.

### Methods for dealing with missing items on the NPQ

One basic issue with the NPQ is that some patients frequently missed Sect. 9, which is relevant to driving. The current study included questionnaires with this missing item, and the patient’s total score was calculated using the formula: Total scores of 8 items / 32 × 100% [13, 16, 18].

### Data analysis

Data analysis was performed using the Statistical Product and Service Solution (SPSS) version 20. The significance level was set at 0.05.

#### Reliability

The reliability of the NPQ-U was assessed by analyzing test-retest reliability and internal consistency as well as measurement errors [30]. To carry out test- retest reliability, 46 randomly selected patients completed the NPQ-U twice with an interval of 48 h to minimize any memory of previous answers and any variations in clinical status. Patients were not given any treatment during this time. The sample size was determined using a power calculation based on previously developed methods to determine the required sample size for a reliability study [31]. Intraclass correlation coefficient (ICC_2,1_) was used to determine test-retest reliability [26, 30, 32]. ICC may vary from 0.00 to 1.00 and the values of 0.60 to 0.80 are considered as good reliability and the value above 0.80 indicates excellent reliability [33]. Cronbach’s alpha was used to calculate the internal consistency [23, 34]. Cronbach’s alpha values between 0.70 and 0.95 are considered to have high internal consistency [35]. The smallest detectable change (SDC) and the standard error of measurement (SEM), which are calculated using the formulas SEM × 1.96 × √2 [35, 36] and standard deviation × √ (1 – ICC) [36], respectively, were used to determine measurement error [32].

#### Content validity

Content validity examines the completeness of item responses, the distribution of the scores, and the magnitudes of ceiling and floor effects [37]. Floor and ceiling effects were considered present if more than 15% of the respondents achieved the highest or lowest possible score [26, 35].

#### Factor analysis

Exploratory factor analysis (EFA) was performed to determine the dimensionality of the items of the questionnaire. To check the appropriateness of the factor analysis Kaiser–Meyer–Olkin’s measure of sample adequacy (KMO) and Bartlett’s test of sphericity were used. Principal component analysis was used as the extraction method with varimax rotation. Using Kaiser’s rule (Eigenvalue greater than 1) and the scree plot, the number of factors retained were determined [29].

Confirmatory factor analysis (CFA) was performed with AMOS software using maximum likelihood estimation to confirm the underlying factor structure from the EFA. Modification indices for the correlation of error terms were determined to improve model fit. The relative chi-square (X^2^/df), standardized root mean square residual (SRMR), root mean square error of approximation (RMSEA) with 90% confidence interval, comparative fit index (CFI), and Tucker-Lewis index (TLI) were used to assess the goodness of fit. The following criteria were used to determine acceptable model fit: X^2^/df ˂ 5, RMSEA ≤ 0.08, SRMR ≤ 0.08, CFI ≥ 0.90, and TLI ≥ 0.90 [38–40]. Hair et al. [41] suggested that model fitness can be decided by at least a minimum of three different indices.

#### Construct validity

Construct validity was determined by using Pearson’s correlation coefficients to calculate the correlation between the NPQ-U and the NPDS, NDI-U and NPRS (convergent validity). Correlation coefficients’ values ranging from 0.00 to 0.09, 0.10–0.39, 0.40–0.69, 0.70–0.89, and 0.90-1.00 indicate a negligible, weak, moderate, strong, or very strong relationship, respectively [42].

Construct validity was also assessed using an independent t-test to determine the difference in total NPQ-U score between patients and healthy participants (discriminative validity).

#### Responsiveness

GROC scale was used to dichotomize the patients in to stable (GROC < 3 to > -3) and improved groups (GROC score ≥ 3) at the end of the treatment [26]. Responsiveness was analyzed by comparing the change scores of the NPQ-U between stable and improved groups through an independent t-test and by correlating the change scores of the NPQ-U with the change scores of the NPDS, NDI-U and NPRS through Pearson’s correlation coefficients [26, 37].

## Results

### Translation and cross-cultural adaptation

There were no significant issues while translating. During the face validity determination, 13 patients did not respond to question 9 (driving). These patients stated that they were unable to answer this question because it was unrelated to their lives. It was decided not to change this section because no modification could solve the problem.

The patients’ overall impression of the NPQ-U was that the instructions and questionnaire items were simple to understand and that they could complete it quickly. They also stated that all of the items are relevant to their condition. As a result, the translated version was finalized without any changes to the original version, allowing the NPQ-U to resume the original version’s concepts and meanings.

### Participant characteristics

The study included 150 NSNP patients and 50 healthy participants with female predominance in each group. Table 1 shows the demographic and clinical characteristics of the participants.

**Table 1:** Participant characteristics

Variables			Patients Group (N = 150) Mean ± SD N/%	Healthy Group (N = 50) Mean ± SD N/%
Age (years)			33.26$\pm$ 12.26	25.68 $\pm$ 8.77
Sex		Male	36/24	17/34
		Female	114/76	33/66
Height (cm)			161.29 $\pm$ 9.02	163.68 $\pm$ 11.07
Weight (kg)			65.87 $\pm$ 14.93	60.56 $\pm$ 12.25
BMI			25.41$\pm$5.78	22.58 $\pm$ 3.86
Duration of neck pain (months)			13.89$\pm$25.34	N/A
NPQ-U (0-100)			29.81 $\pm$23.07	0
NDI-U (0–50)			19.35 $\pm$10.21	0
NPDS (0-100)			50.16 $\pm$21.89	0
NPRS (0–10)			5.43 $\pm$ 2.05	0
Work status	Employed		60/40	9/18
	Un-employed		90/60	41/82
Marital status	Single		61/40.7	40/80
	Married		89/59.3	10/20
Education	Primary		8/5.3	-
	Matric		27/18	3/6
	Intermediate		24/16	12/24
	Graduation		82/54.7	35/70
	Post-graduation		9/6	-

BMI = Body mass index, NDI-U = Urdu version of the neck disability index, NPDS = Neck pain and disability scale, NPQ-U = Urdu version of the Northwick park neck pain questionnaire, NPRS = Numerical pain rating scale

### Reliability

All of the NPQ-U items (ICC_2,1_ = 0.80–0.93) and total scores (ICC_2,1_ = 0.96) had good to excellent test-retest reliability. The Cronbach’s alpha of the NPQ-U was 0.89, indicating that the scale has a high level of internal consistency. The mean and reliability results of the individual items and the total NPQ-U scores are summarized in Table 2.

**Table 2:** Mean and reliability results of the NPQ-U (n = 46)

NPQ-U Score	1st Measurement Mean $\pm$SD	2nd Measurement Mean $\pm$SD	ICC	95% CI	SEM	SDC
Question 1	2.08$\pm$0.82	1.63 $\pm$ 0.79	0.80	0.66–0.88	0.36	1
Question 2	1.49$\pm$0.91	1.41 $\pm$0.93	0.89	0.80–0.93	0.30	0.83
Question 3	0.94 $\pm$0.81	0.93$\pm$ 0.80	0.86	0.77–0.92	0.30	0.83
Question 4	1.83$\pm$ 1.18	2.15 $\pm$1.17	0.87	0.77–0.93	0.42	1.16
Question 5	1.68 $\pm$1.07	1.78 $\pm$ 1.17	0.85	0.75–0.92	0.42	1.16
Question 6	1.84$\pm$ 1.06	1.78 $\pm$ 1.24	0.84	0.73–0.91	0.46	1.28
Question 7	1.57 $\pm$1.01	1.45$\pm$ 1.04	0.90	0.82–0.94	0.32	0.89
Question 8	1.29$\pm$ 0.90	1.26$\pm$ 0.97	0.87	0.78–0.93	0.34	0.94
Question 9	1.60$\pm$ 0.94	1.40 $\pm$ 0.94	0.93	0.88–0.96	0.26	0.72
Total (0-100)	43.51 $\pm$ 19.32	38.40 $\pm$ 19.79	0.96	0.94–0.98	3.88	10.75

NPQ-U = Urdu version of the Northwick Park Neck Pain Questionnaire, ICC = Intraclass correlation coefficient, CI = Confidence interval, SEM = Standard error of measurement, SDC = Smallest detectable change

### Content validity

Section 9, which is about driving, was missed by 70 participants. No floor and ceiling effects on the total score of NPQ-U were present. However, 21% of participants experienced floor effects in the section about pins, needles, or numbness in the arms at night.

### Factor analysis

**Fig. 1:**
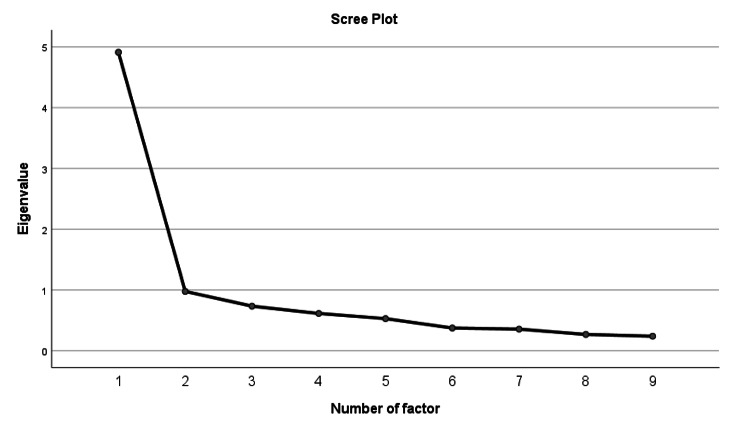
Scree plot showing the one-factor structure of the NPQ-U.

**Fig. 2:**
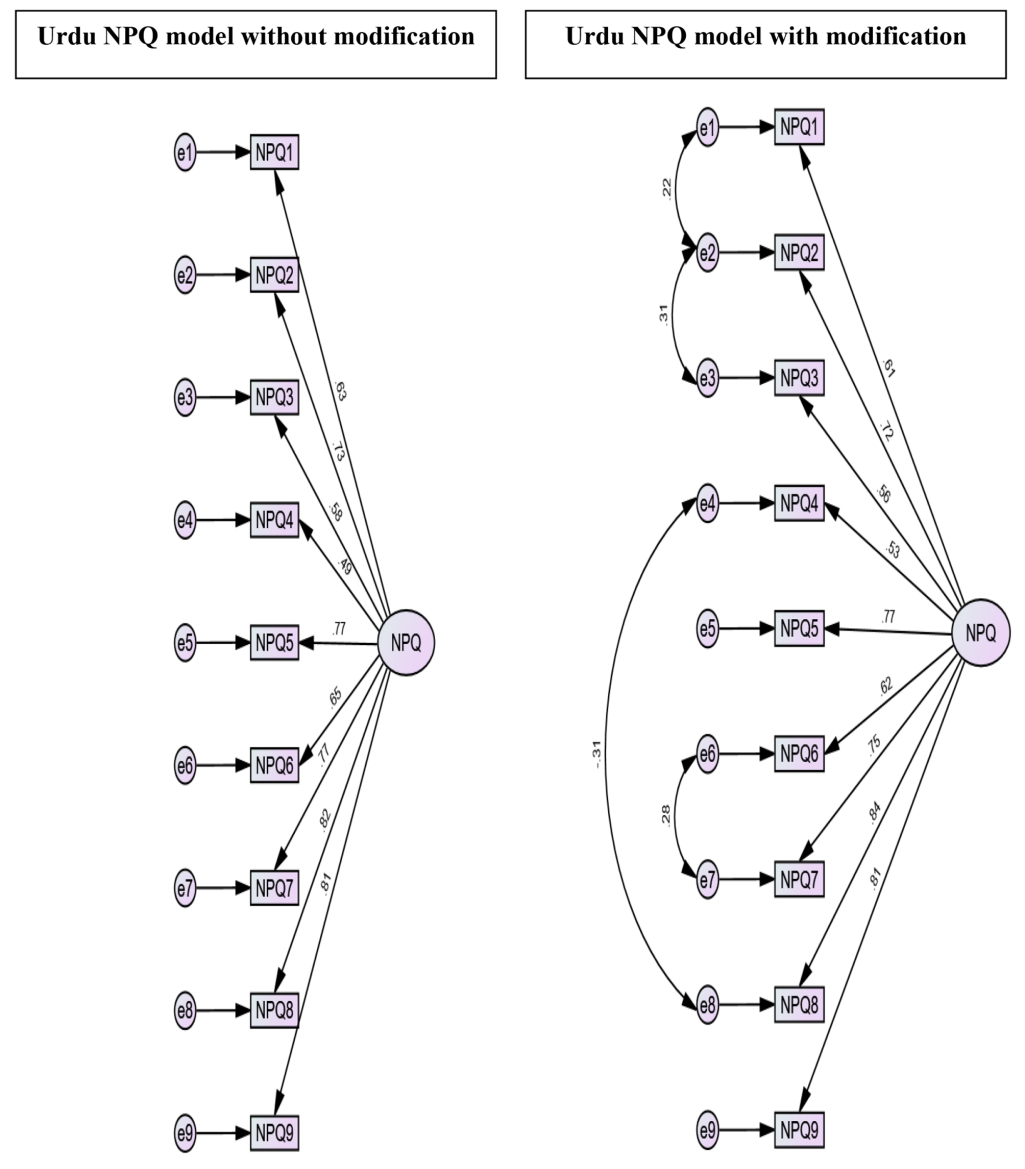
Factor structure of the Urdu NPQ one-factor model. NPQ = Northwick Park Neck Pain Questionnaire

The KMO value was acceptable (0.89), and the Bartlett’s test of sphericity was significant (p < 0.001). As a result, the data was suitable for factor analysis. A 1-factor solution with eigenvalues greater than one was discovered by principal component analysis, accounting for 54.56% of the total variance. A scree plot revealed one factor structure as well (Fig. 1). The CFA confirmed the NPQ-U as a unidimensional scale, as demonstrated by the acceptable model fit indices (Table 3) after allowing four error terms to covary (e1-e2, e2-e3, e4-e8, and e6-e7) (Fig. 2).

**Table 3:** Confirmatory factor analysis of the Urdu Northwick Park Neck Pain Questionnaire one-factor model (n = 150)

Model	X^2^	df	X^2^/df	CFI	TLI	RMSEA (90% CI)	SRMR
Without modification	88.80	27	3.28	0.90	0.87	0.12 (0.096–0.15)	0.06
With modification	46.12	23	2.00	0.96	0.94	0.08 (0.047–0.11)	0.04

X^2^ = Chi-square, df = Degrees of freedom, CFI = Comparative fit index, TLI = Tucker-Lewis index, RMSEA = Root mean square error of approximation, CI = Confidence interval, SRMR = Standardized root mean square residual

### Construct validity

The NPQ-U showed a strong correlation with NDI-U (r = 0.89, P < 0.001), NPDS (r = 0.71, P < 0.001), and NPRS (r = 0.73, P < 0.001). The results revealed a significant difference between patients and healthy controls in the NPQ-U total scores (P < 0.001), demonstrating significant construct (discriminative) validity.

### Responsiveness

The difference in NPQ-U change scores between the two groups was statistically significant (22.06 $\pm$12.37 in the improved group, n = 131; 1.97 $\pm$9.07 in the stable group, n = 19; P < 0.001). The NPQ-U change score had a moderate correlation with NPDS change score (r = 0.60, P < 0.001) and NPRS change score (r = 0.68, P < 0.001), but a strong correlation with NDI-U change score (r = 0.75, P < 0.001).

## Discussion

In this study, the NPQ was first translated and cross-culturally adapted into Urdu, Pakistan’s national language, and then the psychometric testing of the Urdu version of the NPQ was assessed. According to the study findings, the NPQ-U has high reliability, validity, and responsiveness.

The cross-cultural adaptation process was completed according to the predefined guidelines. There were no significant issues while translating. The patients’ overall impression of the NPQ-U was that the instructions and questionnaire items were simple to understand and that they could complete it quickly. As a result, no changes were made to the NPQ-U in order to keep the concepts and meanings of the original version which is in line with earlier studies [15, 16, 18].

In the current study, females (76%) outnumbered males (24%). This is consistent with previous research findings that recruited more females (65.5–82.8%) [13, 15, 16, 18], but it differs from the Korean version of the NPQ, which included more males (56.4%) than females (43.6%) [21]. The patients in this study had a mean age of 33.3 years, which is younger than the average age of participants in previous studies (43.3–55 years) [13, 15–18] but relatively similar to the age of participants recruited in the Brazilian version (35.6) [20].

The NPQ-U demonstrated excellent test-retest reliability in the current study, which is consistent with previous research findings [13, 15, 17–21, 43]. This result demonstrates that the NPQ-U is a scale with a low margin of error in repetitive measurements, producing consistent results from one application to the next. However, the test-retest reliability was higher compared to the Spanish version (0.63) [16]. The lower ICC value in the Spanish version can be attributed to a longer time period (8–10 days) between test and retest. A two-day interval was used in the current study to ensure that no to minimal changes in the patients’ condition occurred. Dawson et al. [44] suggested a 2–3 day interval to avoid major changes in the patients’ conditions. The NPQ-U had excellent internal consistency, with a Cronbach’s alpha of 0.89, as found in previous studies (0.76–0.93) [15, 17, 20, 21, 43].

According to the current study’s findings, a minimum change of 10.75 points on the NPQ-U (0–100 scale) is required to be labeled as a “real change”. In other words, a difference of 10.75 points between total NPQ-U scores before and after treatment was accepted as the threshold value to determine whether there was a clinically significant change without a measurement error. This result is comparable to that of the Brazilian Portuguese version [20].

In the current study, 70 patients (46.7%) did not complete Sect. 9 about driving. Indeed, missing driving data has been reported in Spanish (62.3%) [16], Turkish (69.6%) [15], and French (More than 5%) [18] versions. One explanation for the high percentage of missing responses could be that our patients stated that this item is unrelated to their lives because they do not know how to drive or do not drive despite being in good health. This could also be due to Pakistani women having a lower driving rate than men for socio-cultural reasons. Therefore, we assumed that the patients’ failure to respond to this section was not due to a translation issue, and we did not consider it necessary to make any modifications to this section. Furthermore, this section was not removed because it is relevant for assessing the disability of neck pain [18]. The NPQ-U total scores had no floor or ceiling effects demonstrating excellent content validity, which is consistent with the French version of the NPQ [18].

It is worth mentioning that the NPQ’s factorial structure was not observed in the original English version developed by Leak et al. [13] or in some other translations [15, 16]. In the current study, the EFA of the NPQ-U revealed only one factor which is comparable to what was observed in the Brazilian Portuguese [20] and Hausa [22] versions of the NPQ. The CFA confirmed the NPQ-U as a unidimensional scale after allowing four error terms to covary, which is in line with the findings of the Hausa version [22], which confirmed the NPQ as a unidirectional scale after allowing three error terms to covary. However, in Brazilian Portuguese version, a one-factor model was confirmed by excluding 4 items (i.e., items 1, 3, 4 and 5), which led to a short version consisting of only five items (i.e., items 2, 6, 7, 8 and 9) [20]. The French version of the NPQ, on the other hand, revealed two factors [18]. However, these two factors were not categorized. Pickering et al. [45] also reported a two-factor structure, with the two factors being “dysfunction related to general activities” (factor 1: items 2, 3, 5–9) and “neck pain” (factor 2: items 1 and 4). This variation in results could be attributed to cultural believes about disability, which influence daily living activities.

Due to the lack of a gold standard for health-related questionnaires, the NPQ-U’s criterion validity was not examined [37]. The construct validity of the NPQ-U was found to be good. The NPQ-U showed a positive correlation with the NDI-U, NPDS, and NPRS, consistent with previous studies [15–18, 21, 43]. The effect size of the correlation between NPQ-U and NPRS (r = 0.73) was similar to the findings of the Korean (r = 0.75) [21], Spanish (at re-test) (r = 0.74) [16], and Turkish (r = 0.73) [15] versions but higher than the Argentine (r = 0.66) [43], Chinese (r = 0.58) [17], and French (r = 0.43) [18] versions. The correlation between NPQ-U and NDI-U was strong (r = 0.89) which is quite similar to the findings of the French version (r = 0.88) [18]. Similarly, a strong correlation was found between NPQ-U and NPDS (r = 0.71) which is in line with the findings of French version (r = 0.73) [18]. Furthermore, consistent with the Chinese version of the NPQ [17], the translated version found a significant difference in the NPQ-U total scores between patients and healthy controls. This finding implies that the NPQ-U can differentiate between people who have neck pain and disability and those who do not.

The NPQ-U, as demonstrated by the findings of this study, is an outcome measure capable of detecting changes in condition over time which is comparable with the findings of previous studies [13, 15, 17, 43, 46]. The difference in NPQ-U change scores between the two groups, improved and stable, was statistically significant. This finding is consistent with the results of the French version of NPQ [46]. Additionally, the NPQ-U change scores showed a strong correlation with the NDI-U change scores and a moderate correlation with the NPDS and NPRS change scores. The French version of the NPQ likewise revealed a strong correlation between the NPQ change scores and the NDI change scores as well as a moderate correlation between the NPQ change scores and the NPDS and visual analogue scale for pain change scores [46]. One possible explanation for the NPQ-U’s better correlation with NDI-U scores than with other scales is that these two questionnaires are derived from the Oswestry Disability Questionnaire and have a similar presentation [14]. Similarly, Leak et al. found a significant correlation between changes in the NPQ total scores and the NPQ question 10 score [13]. These findings suggest that the NPQ-U can be used to monitor patients’ improvement.

The current study only included patients with NSNP, and it is unclear whether the results can be applied to patients with other types of neck pain. Furthermore, data were primarily gathered from patients who visited out-patient departments. As a result, the sample might not be a true representation of the general population suffering from neck pain, and the results cannot be generalized to inpatients.

The strength of this study is that the translation and testing of the different psychometric properties of the NPQ-U, including face validity, reliability, content validity, construct (convergent and discriminative) validity, factor analysis, and responsiveness were carried out using standard guidelines. Another strength of the study is that an adequate sample size was used to conduct all of the analyses. In addition, the CFA was used to determine the structure of the NPQ-U. Moreover, though the NDI-U is available to measure disability related to neck pain in Urdu-speaking patients, the NPQ-U appears to have some advantages because it addresses some common daily activities that are likely to be affected by neck pain, such as watching television, carrying objects, and so on, as well as questions about pins and needles or numbness in the arms at night and the duration of symptoms, which the NDI does not. Other than that, the NPQ-U has better psychometric properties to the NDI-U, as its convergent validity and responsiveness were tested against other neck pain and disability scales, such as the NPDS, which the NDI-U lacks. Finally, to the best of the authors’ knowledge, this is the first study that translated and cross-culturally adapted the NPQ into Urdu, as well as tested the psychometric properties of the NPQ-U.

The NPQ-U can be used in clinical and research settings to evaluate patients and monitor the effectiveness of physiotherapy, manipulative therapy, and any other treatment for neck pain to assess disability because of its good psychometric properties and ease of use. Furthermore, it can contribute to more valid cross-cultural comparisons of neck disorders between English and Urdu speaking populations by serving as the primary outcome measure.

## Conclusion

According to the findings of this study, the NPQ-U is a reliable, valid, and responsive questionnaire. The NPQ-U’ items are simple and easy to complete. As a result, it can be used to assess neck pain and disability in Urdu-speaking NSNP patients.

## Data Availability

All data generated or analyzed during this study are included in this published article.

## Abbreviations

BMI: Body mass index
CFA: Confirmatory factor analysis
CFI: Comparative fit index
CI: Confidence interval
df: Degrees of freedom
EFA: Exploratory factor analysis
GROC: Global rating of change scale
ICC: Intraclass correlation coefficient
KMO: Kaiser–Meyer–Olkin’s measure of sample adequacy
NDI-U: Urdu version of the neck disability index
NPDS: Neck pain and disability scale
NPQ: Northwick Park Neck Pain Questionnaire
NPQ-U: Urdu version of the Northwick Park Neck Pain Questionnaire
NPRS: Numerical pain rating scale
NSNP: Non-specific neck pain
RMSEA: Root mean square error of approximation
SD: Standard deviation
SDC: Smallest detectable change
SEM: Standard error of measurement
TLI: Tucker-Lewis index
